# Pigeon during the Breeding Cycle: Behaviors, Composition and Formation of Crop Milk, and Physiological Adaptation

**DOI:** 10.3390/life13091866

**Published:** 2023-09-04

**Authors:** Liuxiong Wang, Jianguo Zhu, Peng Xie, Daoqing Gong

**Affiliations:** 1 College of Animal Science and Technology, Yangzhou University, Yangzhou 225009, China; 15380611359@163.com (L.W.); yzjianguozhu@163.com (J.Z.); 2 Jiangsu Collaborative Innovation Center of Regional Modern Agriculture & Environmental Protection, Huaiyin Normal University, Huaian 223300, China; 3 Jiangsu Key Laboratory for Eco-Agricultural Biotechnology around Hongze Lake, Huaiyin Normal University, Huaian 223300, China

**Keywords:** pigeon, crop milk, behavior, physiological adaptation, prolactin, signaling pathway

## Abstract

Pigeon is an important economic poultry species in many countries. As an altricial bird, its growth and development are largely reliant on pigeon milk produced by the crop tissue in the first week. During the breeding cycle, pigeons undergo a series of behavioral changes. Pigeon milk is generally characterized by having high concentrations of proteins and lipids, and a complicated regulatory network is involved in the milk formation. Hormones, especially prolactin, could promote the proliferation of crop epidermal cells and nutrient accumulation. The expression of target genes associated with these important biological processes in the crop epidermis is affected by non-coding RNAs. Meanwhile, signaling pathways, such as target of rapamycin (TOR), Janus kinase/signal transducer and activator of transcription proteins (JAK/STAT), protein kinase B (Akt), etc., influence the production of crop milk by either enhancing protein synthesis in crop cells or inducing apoptosis of crop epidermal cells. In order to adapt to the different breeding periods, pigeons are physiologically changed in their intestinal morphology and function and liver metabolism. This paper reviews the behaviors and physiological adaptations of pigeon during the breeding cycle, the composition of pigeon crop milk, and the mechanism of its formation, which is important for a better understanding of the physiology of altricial birds and the development of artificial crop milk.

## 1. Introduction

“Pigeon” is the collective name for hundreds of breeds of birds in the *Columbidae* family [1]. As one of the earliest domesticated birds, it is important economically and for entertainment due to its ornamental and racing value, as well as its use for meat and eggs. At present, it has become an ideal animal model for analyzing bird behavior and the physiological regulation of reproduction due to its well-timed breeding cycle and biparental care [2,3].

**Figure 1:**
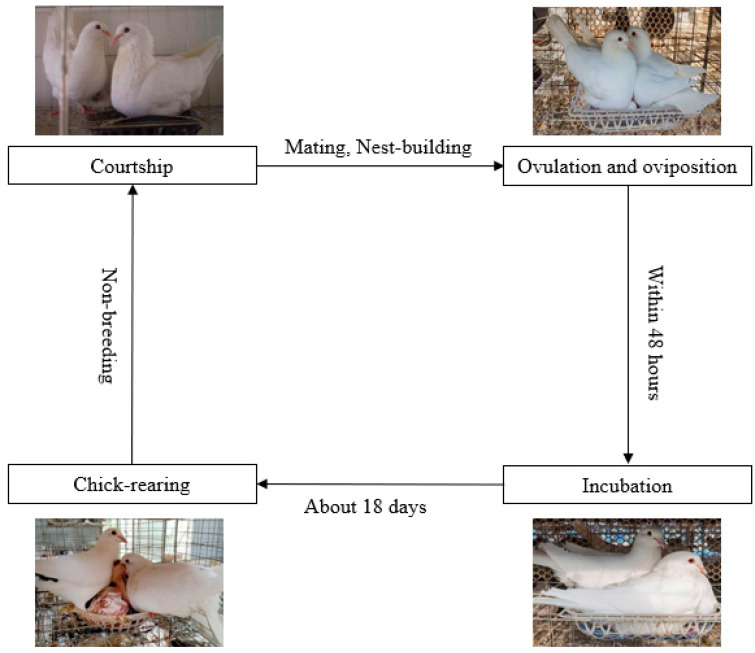
Breeding cycle of pigeons.

Pigeons are non-seasonal breeders, and their breeding cycle includes non-breeding, courtship and mating; nest-site selection and nest building; incubation; and feeding of the squabs [4]. The birds pair off in a pattern of one female and one male for their entire life. The female usually lays two eggs within 48 h, and then the parents take turns to hatch the egg [5]. There is a clear time difference between male and female incubation activities, with males generally incubating at noon and females incubating for the rest of the day [6]. After about 18 days of incubation, the squabs are taken care of by their parents, and the adults regurgitate food to their squabs in a mouth-to-mouth manner (Figure 1) [7,8,9,10]. Similar situations also occur among flamingos and emperor penguins [11,12]. Amazingly, the growth rate of pigeons is much higher than that of other poultry. The relative growth index of squabs is 3.79 and 1.96 times higher than that of large fast-growing chickens and quails, respectively [10].

This paper primarily reviews the behaviors and physiological adaptation of breeding pigeons, the composition of crop milk, and the mechanism of crop milk formation. It will help us to further understand the physiological and behavioral characteristics of altricial birds and could also be of great significance in guiding the development of artificial crop milk.

## 2. Artificial Breeding of Pigeons

In China, the scale of the pigeon industry is steadily increasing year by year [13]. After years of development, pigeons are mainly used for meat products, racing and ornamental purposes [14]. The breeds of meat pigeons include White King, Carnean, Silver King, European meat-type, Tyson pigeon, etc. [15]. The data show that in 2021, the number of breeding pairs exceeded 111 million, and about 1.6 billion pigeon squabs were slaughtered for meat [16]. The price of one pair of breeding pigeons and one squab are about RMB 120–140 and RMB 14–19, respectively [16]. In addition, pigeon-racing (also known as “Duivensport” in Europe) is becoming increasingly popular in many countries [17].

The length of the reproductive use of a breeding pigeon is about 3–5 years. A pair of breeding pigeons can give birth to 10–16 pairs of squabs in one year [18], and a newborn squab usually needs 5–6 months to reach sexual maturity. Research shows that the weight of a newborn squab was positively correlated with the egg weight [19]. Meanwhile, the weight of squabs was also influenced by the breed and care of parental pigeons [20]. The nutrient levels in the diet are closely related to the reproduction performance of breeding pigeons. For instance, the laying rate of pigeons was significantly enhanced by the appropriate levels of dietary lysine, calcium, zinc and crude protein [21,22,23,24].

To increase productivity, newborn squabs are adopted by one pair of breeding pigeons in a “2+3” or “2+4” pattern under artificial farming (3 to 4 squabs nursed by a pair of breeding pigeons) [24]. The body weight and organ development of posthatch squabs were optimal under the feeding system of CWC (whole grains of maize and wheat plus concentrate feed) [25]. In addition, the cafetaria method in intensive rearing influenced the productivity of pigeons [26]. The environment and manual management could affect the ultimate production of pigeon squabs. For example, appropriate intensity and color of light had a positive impact on egg production to indirectly raise the final production of squabs [27,28]. Meanwhile, the egg-laying cycles of pigeons were shorter with egg removal management [29].

## 3. Behaviors during the Breeding Cycle

Both male and female pigeons undergo changes in behaviors that are closely associated with their breeding tasks. At the stage of courtship and mating, the male bird often struts around the female with a bowing posture [30], which is similar to that in ring dove [31]. Sometimes, the male pigeon drives the female and pecks in the open space [30]. Once the female pigeon is successfully attracted, it gently pecks the male at the back of the head when the male is performing nest demonstration [32]. In addition, the female bird also pushes itself under the male body and pecks feathers surrounding the root of the male’s bill [33]. Interestingly, there is a phenomenon occurring during this phase in which the male opens his mouth to let the female’s bill embed into it so that the female can suck food from the crop of the male, which looks similar to the behavior of feeding the young [32]. 

During the nest-site selection and nest-building period, they usually choose a nesting site together. Then, the male bird collects materials to build the nest with the female resting in the nest in preparation for the following copulation and ovulation, which is different from the ring doves in which both the male and female ring doves work together to build the nest [31,32]. After nesting and copulation are performed, the female bird becomes more attached to the nest, which indicates that it is about to lay eggs. At this time, the nest defense behavior in male and female pigeons when they are exposed to danger is avoidance [34]. 

At the incubation phase, both male and female pigeons take a turn to sit on the egg. The male bird often sits on the egg from 10 a.m. to 4 p.m., while the female incubates eggs for the remaining time of each day, which is similar to ring doves [31,35]. The female pigeon invests more time than the male in hatching eggs, which is different from the situation of the nest-building period. At this time, pigeons are very vigilant, and the most predominant form of nest defense behavior is defense, such as feather erection and wing rising [34]. They show aggressive behaviors if they feel that their eggs are in danger. In addition, young breeders that hatch eggs for the first time might fly out of the nest and move freely, resulting in a failure of egg incubation. After approximately 18 days of incubation, parent pigeons might desert the eggs if they find that eggs are not hatched [36]. 

When feeding squabs, both male and female pigeons can produce crop milk. The chicks compete for food with their wings flapping. In addition, squabs receive unparalleled care from parents. For example, one of the parent pigeons always walks around to guard the chicks from danger, such as predators, hot sun, and rain [36]. Parent pigeons at this stage exhibit more aggressive behaviors compared with those in the incubation period, such as pecking and wing slapping, when their babies are in potential danger [34,37,38]. During rest time, the female usually cleans her body and feathers. The parents eat the eggshells after the chicks hatch; thus, the nest is kept sanitary by instinct [39]. After approximately 28 days of the squab-brooding period, squabs are able to eat on their own, and their body weight averages approximately 500 g. At this time, parent pigeons are free of the heavy task of feeding the young and enter into a period of non-breeding.

## 4. The Composition of Pigeon Crop Milk

Bird crops in a low-pH environment created by microbial fermentation act as a functional barrier to pathogens [40,41]. In addition to the function of food storage [8,42], pigeon crops can produce milk. Pigeon milk is the only source of nutrition for chicks in their early growth periods. Research has shown that the 0–3-day-old squab receives crop milk only, and after that, it starts to be mixed with cereals and is gradually replaced by feed [43]. In addition, when the relative content of grain in pigeon milk was elevated, the growth rate of pigeon squabs decreased significantly [44], suggesting that pigeon milk has an irreplaceable role in the rapid early growth of squabs.

There are already some studies on the composition of crop milk, but the results are inconsistent, which may be related to the diversity of feeds taken by parent pigeons and sampling methods [45,46]. As shown in Table 1, pigeon milk is characterized by high concentrations of protein and fat and low concentrations of carbohydrates [47,48,49]. In the first week of crop milk secretion, the constituents of pigeon milk were stable except for protein [44]. There is no doubt that nutrients and even microorganisms in crop milk are essential for the growth and development of squabs.

### 4.1. Protein

Crop milk is rich in proteins. On a dry weight basis, pigeon milk contains approximately 64% protein [50]. Studies have reported that casein accounts for nearly 90% of crop milk protein [51,52]. However, it is well known that casein is a major component of mammalian milk [53,54,55]. Transcriptome analysis identified cornification-associated genes that were differentially expressed in the ‘lactating’ crop [9], suggesting that pigeon milk may be abundant in keratin. The proteomic data of first-week pigeon milk showed that the top 15% of proteins are ribosomal protein, keratin, peroxiredoxin, annexin, heat shock protein and eukaryotic translation protein [56], with no trace of casein. Recently, many types of keratin were found in crop milk, and keratin 4 accounted for the highest proportion [57].

Analysis of the amino acid profile in pigeon milk showed a total of 17 amino acids, with high levels of glutamic acid (Glu), aspartic acid (Asp) and leucine (Leu) and low levels of methionine (Met), tryptophan (Trp), histidine (His) and cysteine (Cys) [58]. Approximately 57.7–59.97% of the total amino acids were composed of 12 essential amino acids during 14 days of secretion [59]. From Day 1 to Day 25 of chick rearing, all essential amino acid and nonessential amino acid contents in pigeon milk decreased significantly [60], which is inconsistent with the results of Zhang et al. [59]. This can be attributed to the different sampling methods. Meanwhile, nearly 17% of the total nitrogen was determined in the form of free amino acids [61].

### 4.2. Lipids

There are rich lipids in pigeon milk. Crop milk consists of 30% fat based on dry weight [50], and its content in both male and female pigeon milk decreased significantly from Day 1 to Day 25 of chick rearing [62]. Notably, the lipid content in male crop milk was significantly higher than that in females on Day 4 of the chick-rearing period, which indicates that its content may be affected by the sex of the parent pigeon [62]. Triglycerides (81.2%) are the main lipid in pigeon milk, followed by phospholipids (12.2%). There are also minor lipid components (each less than 2.5%), including cholesterol, cholesteryl esters, free fatty acids, diglycerides and monoglycerides [63]. Twenty-one kinds of fatty acids were identified in pigeon milk, of which 18:1 fatty acids were the main fatty acids [64]. Furthermore, the most abundant fatty acid in crop milk was oleic acid, which accounted for half of the total fatty acids, followed by linoleic acid and palmitic acid [65].

### 4.3. Carbohydrate

Early studies found that no carbohydrates can be found in pigeon milk [66]. However, Shetty et al. [44,66] determined that pigeon milk contains approximately 0.9–1.5% carbohydrates on a wet weight basis, such as fucose (40%), glucosamine (31%), galactose (12%), mannose (9%) and glucose (8%). Based on dry weight, carbohydrates account for 1–3% of crop milk [8,47]. In addition, the contents of total sugar and reducing sugar in crop milk showed a gradual increase in general from Day 1 to Day 6 of the chick-rearing period based on dry matter [67].

### 4.4. Mineral and Vitamin

The mineral content in crop milk is approximately 5–6%, which is very similar to that of whole cow’s milk [55,68]. Shetty et al. [69] found that the major elements in crop milk included phosphorus (P), calcium (Ca), potassium (K), sodium (Na) and magnesium (Mg), while the trace elements included iron (Fe), zinc (Zn), manganese (Mn) and copper (Cu). In the first week of lactation, the levels of Ca, K, Mg, Na and Mn remained fairly constant, while the levels of P, Fe, Zn and Cu decreased significantly. Compared to cow’s and human milk, pigeon milk definitely possesses higher levels of trace elements, which may also be a factor in the rapid growth of young pigeons [69]. Additionally, pigeon crop milk contains vitamins A, C and B2, but is deficient in vitamin B1; the vitamin B2 content is similar to that of cow’s milk [70].

### 4.5. Active Ingredients

#### 4.5.1. Growth Factor and Immune Active Substance

Eleven-day-old mouse sucklings fed pigeon milk showed a significant increase in the weight of the stomach and distal intestine [71]. DNA synthesis in quiescent Chinese hamster ovary (CHO) cells was enhanced by crop milk supplementation [72]. This indicates the possible presence of growth factors in pigeon crop milk. In human milk, epidermal growth factor (EGF) is the major growth factor. Shetty et al. purified a growth factor from pigeon milk that is similar to epidermal growth factor in mice and found that it could bring about precocious opening of eyelids and eruption of incisors in newborn mice [73,74]. The concentrations of EGF and insulin-like growth factor-1 (IGF-1) in crop tissue homogenates of male and female pigeons were highest on Day 1 of the chick-rearing period [75]. 

The expression of immune-related genes in chickens fed pigeon milk was significantly enhanced [76], which suggests that immune-active substances may exist in crop milk. Additionally, studies have shown that pigeon milk is composed of considerable amounts of immunoglobulin IgA (1.45 mg/mL) and IgG (0.34 mg/mL), which can be transferred to chicks to provide local immunity [77,78]. Pigeon crop milk also includes transferrin and lactoferrin, which possess antimicrobial properties [10,76,79].

#### 4.5.2. Active Enzyme

Various active enzymes are present in pigeon milk, such as aspartate transaminase (AST), alanine transaminase (ALT), alkaline phosphatase (ALP), acid phosphatase (ACP), leucine amino peptidase (LAP), gamma glutamyl transpeptidase (GGTP), trypsin, lipase, amylase, maltase, trehalase, cellobiase, lactase and sucrase [80,81]. They not only assist squabs in digesting nutrients, but also promote the development of their digestive functions. In many mammalian species, the enzyme concentration of colostrum decreases progressively as lactation proceeds [82], while enzyme activities in pigeon crop milk remain fairly stable in the first 4 days of secretion [80].

### 4.6. Microorganism

Pigeon milk was found to be rich in microorganisms, and it contains lactobacilli, streptococci and coliforms [83]. The presence of lactobacilli may be a reason for the acidity of crop milk. Feeding pigeon milk to chickens significantly increased the diversity of cecal microbiota [76], which indicates that many microorganisms in crop milk may contribute to it. Furthermore, the dominant genera of microorganisms in parent pigeon milk are *Lactobacillus*, *Enterococcus*, *Veillonella* and *Bifidobacterium*. Microbial functional analysis showed that these microbes were involved in the pathways of carbohydrate metabolism, amino acid metabolism, and energy metabolism [1]. This suggests that microbiota in pigeon crop milk may play an important role in many aspects, such as helping the digestion of squabs, maintaining gut homeostasis of hosts, facilitating postnatal development and enhancing immune function [84,85,86].

## 5. The Formation Mechanism of Pigeon Milk

**Figure 2:**
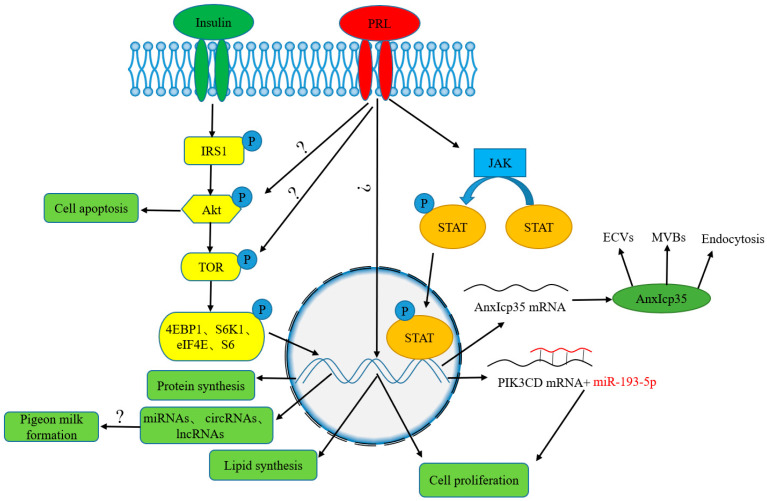
The regulatory mechanism of pigeon milk formation. The symbols “?” in this figure indicates that these associated pathways are not clarified.

The formation of pigeon milk is involved in a very complex regulatory network, as shown in Figure 2. Two biological processes are involved. One is the proliferation and shedding of crop epidermal cells, and the other is the accumulation of nutrients in cells. Under the stimulation of hormones, especially prolactin, crop cells proliferate massively, and the proliferation of the crop epidermis is also affected by non-coding RNAs. Meanwhile, a large number of nutrients, such as proteins and lipids, are synthesized in crop epidermal cells. When nutrients have accumulated to a certain level, apoptosis of epidermal cells occurs, which leads to the shedding of a mass of epithelial cells that are full of nutrients to form pigeon milk.

### 5.1. The Proliferation of Pigeon Crop Epidermal Cells

#### 5.1.1. Morphological Changes in the Crop

In birds, the crop is located between the distal esophagus and the proximal end of the proventriculus. In the nonlactating phase, the crop wall of pigeons is thin, and there is no lateral lobe structure [87]. In preparation for lactation, the number and depth of rete pegs increases significantly and the lamina propria becomes progressively more extended and narrow as the crop further differentiates [9]. This largely increases the surface area of the crop to provide the histological basis for the mass production of crop milk. During the lactation period, in response to prolactin, the rapid proliferation of the germ cell layer of crop tissue leads to a convoluted, highly folded epithelial structure [88,89]. The whole crop wall is thickened with two obvious lateral lobe structures, and its weight and volume are significantly enlarged [8,87]. The nutritive cell layer is then formed as the vasculatures emerge. Finally, the nutrient-loaded cells are shed to produce crop milk [9].

#### 5.1.2. The Regulation of Hormones on Crop Proliferation

Prolactin plays an important role in the regulation of reproduction in vertebrates. Riddle et al. [90] first discovered and purified prolactin from the anterior pituitary of pigeons. The level of prolactin rose in adult pigeons during the latter half of the incubation period, and the development of the crop sac directly followed increased hormone secretion [55]. Injection of exogenous prolactin significantly increased crop weight [91,92] and levels of DNA and RNA [92], and it also induced expression of specific genes [92,93,94]. There are at least two modes by which prolactin acts as a mitogen on the epithelium of crop: First, prolactin acts directly on the crop mucosa to enhance the sensitivity of the crop to a somatomedin-like growth factor that functions to promote mitosis; second, prolactin heightens the synthesis and secretion of somatomedin-like growth factor, which then acts synergistically with prolactin to stimulate the proliferation of crop mucosa cells [95]. Studies have shown that ornithine decarboxylase activity is closely related to cell proliferation [96,97]. Prolactin increased ornithine decarboxylase activity and promoted DNA synthesis and cell proliferation in human promyelocytic cells [98]. Likewise, prolactin injection significantly increased ornithine decarboxylase (ODC) activity in pigeon crop [92]. Therefore, the rapid proliferation of the crop during lactation may be related to the increased activity of ornithine decarboxylase induced by prolactin stimulation.

Relaxin is mainly produced by the corpus luteum and attains high plasma levels during pregnancy [99]. Studies have shown that relaxin could cause significant development of the mammary gland in rats [100,101,102]. Likewise, relaxin was reported to enhance the cell growth and differentiation of the crop sac, which induces a significant increase in crop weight [103,104]. Bani et al. [105] also found that the changes in the crop sac following prolactin action were similar to those following relaxin injection. Therefore, relaxin may be involved in pigeon milk formation by promoting crop proliferation. Instead, estradiol, progesterone and growth hormone may not contribute to crop milk formation [75]. In addition, the concentrations of EGF and IGF-1 in pigeon crop homogenates were notably elevated during the breeding period [75], and injection of EGF significantly enhanced the local growth of pigeon crop-sac mucosal epithelium [106]. Thus, EGF may also be involved in crop milk formation.

#### 5.1.3. The Regulation of Crop Proliferation by Non-Coding RNA 

Regulatory non-coding RNAs consist of small non-coding RNAs (small ncRNAs) and long non-coding RNAs (lncRNAs). Small non-coding RNAs include circular RNAs (circRNAs), microRNAs (miRNAs), and small interfering RNAs (siRNAs). Studies have shown that regulatory non-coding RNAs regulate mammary gland development and proliferation in mammals [107,108]. For example, circHIPK3 promoted mammary epithelial cells proliferation in bovine [109]. Comparing the expression profiles of miRNAs in the crop of lactating and nonlactating female pigeons, a total of 71 miRNAs were significantly differentially expressed. The target genes of these miRNAs were mainly involved in cell development and epithelial cell morphogenesis, which indicates that these miRNAs could regulate crop cell proliferation to affect pigeon milk formation [110]. In addition, miR-193-5p bound to a conserved site in the 3′-untranslated region (UTR) of phosphatidylinositol-4,5-bisphosphate 3-kinase catalytic subunit delta (PIK3CD) mRNA to negatively regulate its expression to promote the proliferation of pigeon crop fibrocyte [111].

### 5.2. Accumulation of Nutrients in Pigeon Crop Epithelial Cells

#### 5.2.1. Synthesis of Protein in Crop Epidermal Cells

Amino acids are the building blocks in the process of protein synthesis that control various metabolic pathways important for whole-body homeostasis [112]. During lactation, the process of amino acid uptake from circulation into the mammary gland was strengthened [113], so amino acid concentrations in plasma are often considered an indicator of milk protein synthesis [114]. Xie et al. [60] found high levels of essential and non-essential amino acids in plasma on Day 1 or 7 of the chick-rearing period, and the expression of amino acid transporters and enzymes related to amino acid synthesis in the crop tissue was also higher at this point in time. This suggests that both amino acid transportation and de novo synthesis are necessary for the synthesis of proteins in crop cells.

Under the stimulation of prolactin and insulin, a series of signaling pathways were activated to enhance protein synthesis in crop epidermal cells, such as the target of rapamycin (TOR), Janus kinases/signal transducer and activator of transcription proteins (JAK/STAT), and insulin receptor substance 1/protein kinase B/target of rapamycin (IRS1/Akt/TOR) signaling pathway. In mammals, the mammalian target of rapamycin (mTOR) signaling pathway has been shown to play an important role in the synthesis of milk proteins by changing the phosphorylation status of downstream effector proteins, such as eukaryotic initiation factor 4E binding protein 1 (4EBP1) and ribosomal protein S6 kinase (70S6K) [115,116,117,118,119]. Targeted disruption of the target of rapamycin complex 1 (TORC1) inhibited protein synthesis in pigeon crop, thereby retarding the growth of squabs [120]. Dietary supplementation with leucine promoted the growth of squabs and increased the synthesis of pigeon milk protein through the TOR signaling pathway [51], which is consistent with that in mammary glands [121]. Meanwhile, DL-methionine or DL-methionine-DL-methionine supplementation to the parent diet significantly enhanced protein synthesis in crop tissue by the JAK2/STAT5 signaling pathway [52]. In addition, the synthesis of protein in crop epidermal cells was also enhanced through activation of the IRS1/Akt/TOR signaling pathway [50]. The avian prolactin receptor consists of two repeats of the extracellular ligand-binding domain. Binding of prolactin to its receptor leads to phosphorylation of one or more STAT proteins through activation of JAK [52,55]. Then, the STAT protein is translocated into the nucleus and interacts with sites of the anxIcp35 gene and other potential genes to regulate their transcription. The anxIcp35 protein was engaged in the formation and trafficking of endocytotic vesicles (ECVs) and multivesicular bodies (MVBs), which are very important for the uptake of extracellular nutrients [55,122,123]. At the same time, anxIcp35 is also involved in protein synthesis in pigeon crop [94,124]. 

In the mammary gland of goats, the AMP-activated protein kinase-mammalian target of rapamycin (AMPK-mTOR) pathway participates in the sensing and utilization of amino acids [125]. A total of 770 differentially expressed circRNAs (DECs) were identified in lactating and nonlactating crops. The Kyoto Encyclopedia of Genes and Genomes (KEGG) pathway enrichment analysis showed that the DECs were enriched in the AMPK signaling pathways [126], which suggests that circRNAs may be associated with protein synthesis in crop tissue. The target genes of miRNAs that include miR-20b-5p, miR-146b-5p, miR-21-5p and miR-26b-5p were also engaged in the processes of protein synthesis, uptake and digestion [110]. Furthermore, KEGG analysis of 6166 differentially expressed lncRNAs in pigeon crop showed that the biosynthesis of amino acids was significantly enriched [127].

#### 5.2.2. Synthesis of Lipid in Crop Epidermal Cells

Lipids in crop milk are regarded as an important source of energy for the growth and development of pigeon squabs. In the mammary gland of mammals, lipoprotein lipase was significantly upregulated in the de novo synthesis of triglycerides [128,129,130]. Injection of prolactin into the crop of adult pigeons significantly increased the activity of lipoprotein lipase [131]. In mammals, de novo lipogenesis requires the activity of key enzymes, including acetyl-CoA carboxylase (ACC) and fatty acid synthase (FAS) [132]. During the peak time of lactation, the mRNA expression of ACC and FAS in pigeon crop showed high levels [62]. In addition, thirty-four genes associated with lipid synthesis were differentially expressed in the lactating crop. Among them, Elovl6 was significantly upregulated in the lactating pigeon crop [9], which has high elongation activity on C16:0 long-chain fatty acids and some activity on C18:1 and C18:2 long-chain fatty acids [133].

The entry of exogenous long-chain fatty acids into epithelial cells of the mammary gland relies on special transport systems, including fatty acid translocase (FAT/CD36), fatty acid transport proteins and lipoprotein lipase, etc. [134,135]. The expression of FAT/CD36, fatty acid-binding protein 5 (EFABP), and acyl-CoA-binding protein (ACBP) in male pigeon crop reached a maximum on Day 17 of incubation [62]. However, in female crop, the mRNA expression levels of FAT/CD36, EFABP and ACBP were highest on Day 14 of incubation [62], suggesting that female pigeons were prepared earlier for lipid synthesis than males. This indicates that fatty acids used for lipid biosynthesis in crop epidermal cells probably originated from exogenous supply at the terminal phases of incubation. At the same time, lipid accumulation in crop epidermal cells induced oxidative stress in mitochondria to inhibit the process of fatty acid β-oxidation, which could, in turn, increase fat deposition [136]. 

In mammals, regulatory non-coding RNAs can regulate milk fat synthesis. For example, several important circRNAs affecting dairy milk fat synthesis were identified, including circ_0001122, circ_0007367, circ_0018269, and circ_0015179 [137]. Additionally, downregulation of miR-29s significantly inhibited the synthesis and secretion of triglycerides in cultured dairy cow mammary epithelial cells [138]. Likewise, Ge et al. determined that miR-20b-5p, miR-146b-5p, miR-21-5p and miR-26b-5p were key miRNAs and found that the target genes of these miRNAs were engaged in lipid synthesis in pigeon crop cells [110]. Although the differential expression profiles of non-coding RNAs in lactating and nonlactating pigeon crops have been revealed [110,126], the mechanism by which these non-coding RNAs regulate lipid synthesis in crop cells remains to be further investigated.

#### 5.2.3. Synthesis of Carbohydrates in Crop Epidermal Cells

Although carbohydrates are very important for the growth and development of birds [139,140], their contents in pigeon milk are very low. Zhu et al. [67] showed that the mRNA expression level of sodium-dependent glucose transporter 1 (SGLT1) in the crop of male and female pigeons was significantly suppressed from Day 17 of incubation to Day 7 of the chick-rearing period. Similarly, glucose transporter 2 showed the lowest mRNA expression level on Day 17 of incubation [67]. AMP-activated protein kinases (AMPKs) are important cellular energy sensors that regulate glucose metabolism by promoting glucose uptake [141,142]. During the peak of lactation, the expression levels of AMPK signaling pathway-related proteins were significantly inhibited [67]. These results suggest that the process of glucose uptake by pigeon crop was severely restricted, thus resulting in a very low carbohydrate content in pigeon milk.

### 5.3. Shedding of Crop Epidermal Cells

Cell apoptosis is an indispensable factor that finally contributes to the shedding of crop epidermal cells to produce milk [143]. As mentioned above, many nutrients, such as proteins and lipids, are synthesized in the process of crop milk formation. Excessive fat deposition in the mammalian liver leads to lipoapoptosis, fibrosis, and steatohepatitis [144]. In pigeon crop cells, the oxidative stress of mitochondria was brought about by lipid overaccumulation, which ultimately results in apoptosis [136]. The Ca^2+^ channel plays an important role in the exchange of Ca^2+^ between the endoplasmic reticulum and mitochondria [145]. When unfolded or misfolded proteins accumulate excessively in cells, endoplasmic reticulum stress (ERS) occurs [146]. ERS induced a large influx of Ca^2+^ into mitochondria, causing its stress to evoke cell apoptosis [147]. During lactation, the expression of genes related to amino acid transportation and de novo synthesis was significantly elevated in crop tissue [60]. This indicates that crop milk protein is synthesized and accumulates massively in epidermal cells, which may induce ERS to stimulate their apoptosis. In addition, the expression levels of apoptosis-related genes in crop tissue were highest around Day 17 of incubation and Day 1 of the chick-rearing period [143], suggesting that the crop epidermis undergoes a drastic apoptotic response during the formation of pigeon milk.

## 6. Physiological Adaptation in Pigeons during the Breeding Period

### 6.1. Changes in Intestine Morphology and Function

The intestine is a major site of digestion and absorption. Intestinal functions are reflected by intestinal morphology and structure, such as villus height, crypt depth and surface area [148]. Although the villus height of the intestine was stable in adult pigeons, the crypt depth and surface area of the intestine showed a significant increase from the terminal phase of incubation to the early phase of the chick-rearing period [149]. This may be related to increased food intake and high production of crop milk. The expression level of nutrient transporters, including fatty acid transporters, amino acid transporters, glucose transporters and oligopeptide transporters, showed dynamic changes during the incubation and chick-rearing periods [149], which indicates that the development of the intestine is notably affected by different breeding stages. Digestive enzymes are of great importance for the digestion function of the small intestine. In rats, the activities of digestive enzymes increased during pregnancy and lactation [150]. Likewise, the activities of the digestive enzymes Na^+^-K^+^ ATPase and Aminopeptidase-N in the duodenum and maltase in the jejunum were also significantly affected by breeding stages [149]. This suggests that intestinal function undergoes significant changes to adapt to the different breeding periods.

### 6.2. Changes in Liver Metabolism

In mammals, a variety of studies have suggested that the adjustments of liver function from pregnancy to lactation are associated with metabolic pathways that include changes in hepatic gene expression and key enzyme activity [151,152]. Wan et al. [153] investigated glucose and lipid metabolism-related parameters in pigeon liver and found that the process of glucose and lipid metabolism was enhanced from the terminal phase of incubation to the mid phase of chick-rearing. This indicates that the shift in period affects the metabolic status of the liver. The transition from one period to another period in pigeons could cause physiological changes, such as changes in serum biochemical parameters and hormone levels [154]. Serum biochemical parameters can reflect liver function, such as aminotransferase activity and protein, triglyceride, and uric acid levels [155]. During incubation and chick-rearing periods, the profile of serum biochemical parameters in pigeons showed dynamic changes, of which total protein, albumin, globulin, triglyceride, total cholesterol and low-density lipoprotein showed the highest concentrations on Day 17 of incubation [75]. In addition, the contents of serum albumin, total protein and calcium increased significantly from the egg-laying stage to the incubating stage. These results suggest that pigeons at different breeding periods adapt to physiological changes by regulating liver metabolism.

## 7. Can Artificial Crop Milk Be Successful?

In recent years, the market demand for meat pigeons has risen rapidly. Pigeon squabs are altricial birds, which largely restricts the production efficiency of meat pigeons and leads to enormous economic loss. Therefore, the development of artificial alternatives to natural pigeon milk for feeding chicks during the rearing period can be a good solution. However, there are few studies on the development of artificial crop milk.

The composition of natural pigeon milk during the early growth period is very complex, containing not only high protein and lipid contents, but also some enzymes, minerals, immunoglobulins and unknown growth factors, making it very difficult to develop artificial substitutes for crop milk. Limited studies have reported the nutrient requirements of pigeon squabs. The growth performance of 0–7-day-old pigeon squabs was optimum when fed a mixture diet with a metabolic energy of 15.38 MJ/kg and 53.3% crude protein [45]. Feeding artificial crop milk to squabs at the early growth stage resulted in unsatisfactory growth performance compared with that of squabs under the natural feeding mode [8,156]. In the middle and late stages of growth, more cereal grains appear in the crop milk, which reduces the difficulty of developing artificial pigeon milk to some degree. A diet providing 13.04 MJ/kg and 17.77% crude protein could significantly promote the weight gain of 7–25-day-old pigeon chicks, but their growth performance was still lower than that of squabs fed by parent pigeons [157]. Additionally, research on facilities for feeding pigeon chicks is still inadequate and is also constrained for the application of artificial crop milk.

## 8. Conclusions and Prospect

During the breeding cycle, pigeonss undergo a series of behavioral and physiological changes to adapt to the transition period. Nutritive pigeon milk is very important for the growth and development of squabs. The formation of pigeon milk involves a complex regulatory mechanism. Under the stimulation of hormones and other factors, the crop tissue changes dramatically in morphology, and nutrients in crop epidermal cells accumulate rapidly. The crop epidermal cells are finally sloughed off to produce milk, in which intense cell apoptosis is involved. Research on the mechanism of pigeon milk formation would help to further understand the physiology of altricial birds. At present, the growth performance of squabs fed with artificial pigeon milk cannot achieve the same results as those fed naturally, but it is undeniable that the development of artificial pigeon milk still has a bright future for its potential benefits.

## Figures and Tables

**Table 1:** The main composition of pigeon milk and their proportion.

Composition	Proportion (Based on Dry Weight)
Protein	about 64%
Lipid	about 30%
Mineral	about 5–6%
Carbohydrate	about 1–3%

## Data Availability

No applicable.
